# Supplementary material for: Splice-Junction-Based Mapping of Alternative Isoforms in the Human Proteome
Source: Cell Rep. Author manuscript; Available in PMC 2020 Jan 15. (PMC6961840; doi:10.1016/j.celrep.2019.11.026)

A

sp|Q9Y5Y2|NUBP2_HUMAN|ENSG00000095906|SE1|47264|chr16|1783036|1785902|+0|r103|T1
TLEEGHDFIQEFPGSPAFAALTSIAQKILDATPACLMEAAAGVWG q value: 0.00049023 Tr_novel:TRUE RefSeq_Novel:
Search result spec prec mz: 1184.0951 Actual spec prec mz: 1184.0951
Fragments matched per AA: 1.07 Proportion of top 20 peaks matched: 0.2

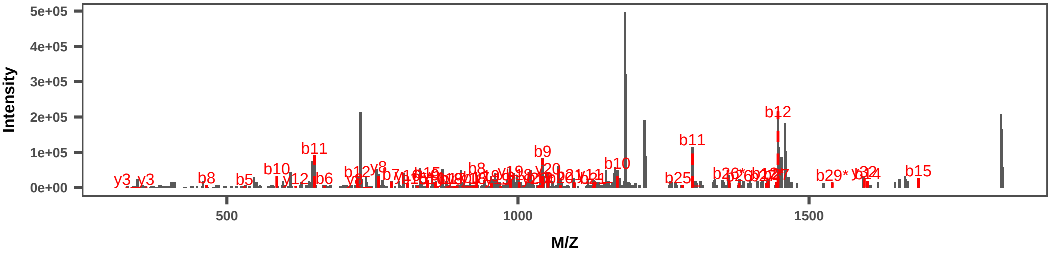

B

Scatterplot of predicted elution time
Fitting R2: 0.845
Novel peptide residual Z score: −1.47
Number of peptides: 1723

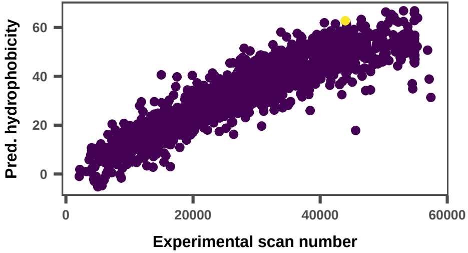

C

Distributions of residuals from best−fit line
of predicted RT vs Expt. scan number
Line: Z score of novel peptide
Z: −1.47

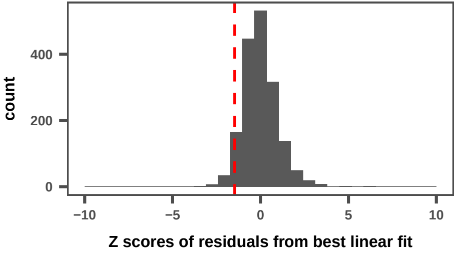

Supplement: 2 [file NIHMS1546469-supplement-2.zip › DF1/PXD010154/Heart/Heart_64_NUBP2_TLEEGHDFIQEFPGSPAFAALTSIAQKILDATPACLMEAAAGVWG.pdf]
